# Study on Vibration Characteristics of Functionally Graded Material Composite Spherical Piezoelectric Transducer

**DOI:** 10.3390/s25051514

**Published:** 2025-02-28

**Authors:** Sha Wang, Junjie Shan

**Affiliations:** 1 Ocean College, Jiangsu University of Science and Technology, Zhenjiang 212100, China; 2 Key Laboratory of State Manipulation and Advanced Materials in Provincial Universities, School of Physics and Technology, Nanjing Normal University, Nanjing 210023, China

**Keywords:** spherical piezoelectric transducer, electromechanical equivalent circuit method, vibration characteristics, functionally graded material

## Abstract

Non-uniform composite structures for transducers exhibit considerable potential in enhancing impedance matching and efficiency. Here, a functionally graded material composite spherical piezoelectric transducer (FGM-cSPT) is proposed, and a three-port electromechanical equivalent circuit model is established. The correctness of the theoretical model is verified using the finite element method and experiment. Based on the theoretical model, the effects of the non-uniform coefficient and the geometric dimension of FGM-cSPT on the electromechanical vibration characteristics (resonance frequency, anti-resonance frequency, and effective electromechanical coupling coefficient) of the transducer are analyzed. The results show that the non-uniform coefficient and geometric dimension can effectively regulate the vibration characteristics of the FGM-cSPT, which can be used to guide engineering design. Our methodology will offer possibilities for designing FGM-cSPTs and may promote applications in various fields, such as marine exploitation, structural health detection, and energy collection.

## 1. Introduction

Spherical piezoelectric transducers are the optimal choice to realize omnidirectional radiation, which is widely used in marine exploitation, structural health detection, and energy collection [1,2,3]. Spherical piezoelectric transducers are usually composite metals to enhance structural strength and improve impedance matching. Wang et al. proposed a sandwiched spherical piezoelectric transducer to enhance the heat dissipation capacity of the spherical transducer through composite metal [4,5]; Tang et al. proposed a cascade spherical piezoelectric transducer to increase the electrical power of the spherical piezoelectric transducer [6,7]; and Kong et al. presented a novel spherical piezoceramic transducer called a spherical smart aggregate, which can be embedded in concrete structures for health monitoring [8,9].

On the other hand, non-uniform composite piezoelectric transducers have shown great potential in improving the performance of transducers in recent years. For example, Dong et al. designed a metagel impedance transformer to achieve broadband transmission, which is inspired by the gradient acoustic impedance distribution in the head of an Indo-Pacific humpback dolphin [10]. Bian et al. filled a specific binary mixture into the conical cavity structure produced by 3D precise printing to build a matching layer where the acoustic impedance decreases exponentially along the thickness direction, which can make the transmitter voltage response bandwidth reach 110% [11]. Ji et al. developed an acoustic impedance matching layer that achieves a gradient variation in equivalent sound impedance in the thickness direction, and its lateral structure is designed as a phononic crystal, which can effectively combine acoustic impedance matching and lateral vibration suppression [12]. Non-uniform composite structures can improve the performance of transducers in terms of impedance matching and efficiency. The material parameters of functionally graded material (FGM) show gradient changes that are the same as those of non-uniform graded materials mentioned in the literature. Therefore, using FGM as composite layers is also expected to achieve the function of non-uniform composite layers [13,14,15].

Nevertheless, research on non-uniform composite structures of spherical piezoelectric transducers, particularly their vibration characteristics, still needs to be completed. Consequently, there is a compelling need to develop theoretical models for acoustic vibration analysis of such transducers. Among the well-established methodologies, the finite element method (FEM) is a reliable and effective approach [16,17,18]. However, this method is computationally intensive, and extensive calculations are time-consuming. Therefore, it is of great significance to propose a simplified and convenient theoretical model. The electromechanical equivalent circuit method (EECM) has straightforward physical significance, and can predict the vibration characteristics of piezoelectric transducers. The elements based on the EECM can be connected by boundary conditions, which are a valuable tool for analyzing and optimizing transducer performance [19,20]. The EECM can analyze more complex vibration systems based on the characteristics linked by boundary conditions. Due to the principle of the electric force analogy, the electromechanical equivalent circuit method can analyze the impedance characteristics of piezoelectric transducers, and is often used to analyze the resonance frequency, anti-resonance frequency, and effective electromechanical coupling coefficient of piezoelectric transducers. The EECM can be used for the preliminary design of piezoelectric transducers [21,22,23].

In this study, the EECM is proposed to analyze vibrational characteristics of a functionally graded material composite spherical piezoelectric transducer (FGM-cSPT). Based on this model, the resonance frequency, anti-resonance frequency, and the effective electromechanical coupling coefficient of FGM-cSPT are investigated. The theoretical results are subsequently validated through experimental results, lending credibility to the analysis.

## 2. Structure and Principle

### 2.1. Structure of FGM-cSPT

**Figure 1:**
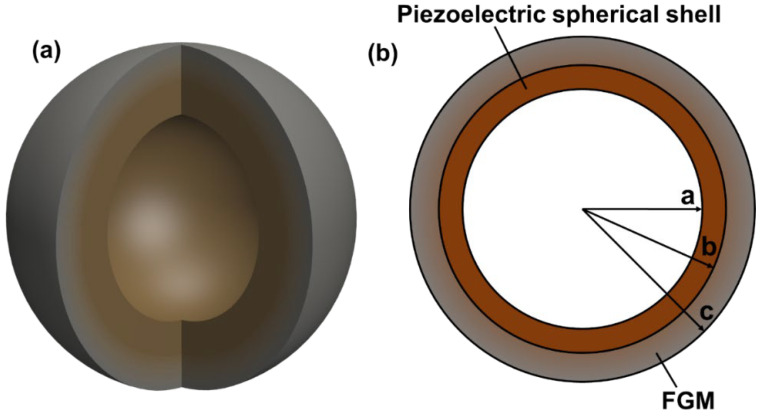
Diagram of FGM-cSPT (**a**) Three-dimensional diagram (**b**) Two-dimensional cross-section.

The FGM-cSPT is composed of a spherical piezoelectric ceramic and FGM spherical shell, as shown in Figure 1. The inner radius of the piezoelectric ceramic spherical shell is “*a*” and the outer radius is “*b*”. The FGM spherical shell is composed of a ceramic–hydrogel composite, and its mixing ratio varies exponentially along the radial direction. The inner radius is *b*, the outer radius is *c*, and the distribution law of Young’s modulus (*E*), density (ρ), and Poisson’s ratio (ν) is presented as follows,

(1) $$E=E_{p}+\left(E_{W}-E_{P}\right){\left(\frac{r-b}{c-b}\right)}^{\beta },$$

(2) $$\rho =\rho_{p}+\left(\rho_{W}-\rho_{P}\right){\left(\frac{r-b}{c-b}\right)}^{\beta },$$

(3) $$\nu =\nu_{p}+\left(\nu_{W}-\nu_{P}\right){\left(\frac{r-b}{c-b}\right)}^{\beta },$$

where $E_{p}$, $\rho_{p}$, and $\nu_{p}$ are, respectively, Young’s modulus, density, and Poisson’s ratio of ceramics, $E_{W}$, $\rho_{W}$, and $\nu_{W}$ are, respectively, Young’s modulus, density, and Poisson’s ratio of hydrogels, and *β* is non-uniform coefficients.

### 2.2. EECM for FGM-cSPT

**Figure 2:**
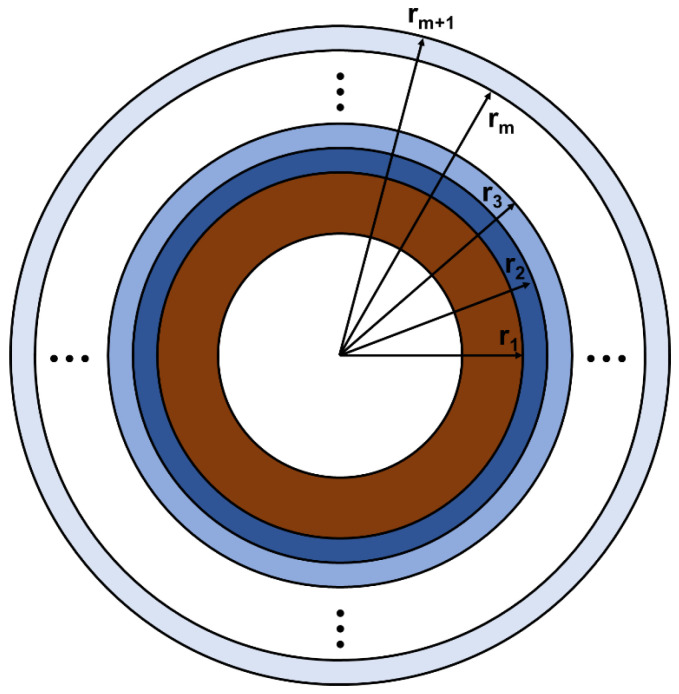
Diagram of FGM-cSPT based on “m” sub-spherical shells.

Unlike the exponential change of a single material, obtaining an analytical solution for FGM with a transition change between two materials is difficult. An approximate laminate model is a common and effective method to simulate a functionally graded sphere with arbitrary radial material properties [24,25]. Therefore, the FGM spherical shell is divided into “m” sub-spherical shells (m layers), assuming each sub-spherical shell is isotropic, as shown in Figure 2.

Therefore, the material parameters of layer i are shown as follows,

(4) $$E_{i}=E_{p}+\left(E_{W}-E_{P}\right){\left[\frac{it}{c-b}\right]}^{\beta },$$

(5) $$\rho_{i}=\rho_{p}+\left(\rho_{W}-\rho_{P}\right){\left[\frac{it}{c-b}\right]}^{\beta },$$

(6) $$\nu_{i}=\nu_{p}+\left(\nu_{W}-\nu_{P}\right){\left[\frac{it}{c-b\;}\right]}^{\beta },$$

where $t=\frac{c-b}{m}$, i = 1,2……m.

The force and vibration velocity of each layer are continuous at the interface. According to the boundary conditions of each layer of the isotropic spherical shell at the interface, the EECM as shown below can be obtained.

The impedance expression of each layer is shown below [4],

(7) $$\begin{array}{c} Z_{i1}=\left(2c_{12(i)}-{\frac{1}{2}c}_{11(i)}-\alpha (i)c_{11(i)}\right)\frac{4\pi r_{i}j}{\omega }\\ \;+\frac{c_{11(i)}r_{i}^{2}4\pi jq_{\left(i\right)}}{\omega \Lambda_{1(i)}}\left[\begin{array}{c} J_{\alpha (i)}\left(q_{\left(i\right)}r_{i+1}\right)Y_{\alpha (i)-1}\left(q_{\left(i\right)}r_{i}\right)\\ -J_{\alpha (i)-1}\left(q_{\left(i\right)}r_{i}\right)Y_{\alpha (i)}\left(q_{\left(i\right)}r_{i+1}\right) \end{array}\right]\\ \;-c_{11\left(i\right)}\sqrt{r_{i}}\sqrt{r_{i+1}}\frac{8j}{\omega \Lambda_{1\left(i\right)}}, \end{array}$$

(8) $$\begin{array}{c} Z_{i2}=\left(-2c_{12(i)}+{\frac{1}{2}c}_{11(i)}+\alpha (i)c_{11(i)}\right)\frac{4\pi r_{i+1}j}{\omega }\\ +\frac{c_{11(i)}r_{i+1}^{2}4\pi jq_{\left(i\right)}}{\omega \Lambda_{1(i)}}\left[\begin{array}{c} J_{\alpha (i)}\left(q_{\left(i\right)}r_{i}\right)Y_{\alpha (i)-1}\left(q_{\left(i\right)}r_{i+1}\right)\\ -J_{\alpha (i)-1}\left(q_{\left(i\right)}r_{i+1}\right)Y_{\alpha (i)}\left(q_{\left(i\right)}r_{i}\right) \end{array}\right]\\ -c_{11\left(i\right)}\sqrt{r_{i}}\sqrt{r_{i+1}}\frac{8j}{\omega \Lambda_{1\left(i\right)}}, \end{array}$$

(9) $$Z_{i3}=c_{11(i)}\sqrt{r_{i}}\sqrt{r_{i+1}}\frac{8j}{\omega \Lambda_{1(i)}},$$

where $\Lambda_{1(i)}=J_{\alpha (i)}\left(q_{\left(i\right)}r_{i+1}\right)Y_{\alpha (i)}\left(q_{\left(i\right)}r_{i}\right)-J_{\alpha (i)}\left(q_{\left(i\right)}r_{i}\right)Y_{\alpha (i)}\left(q_{\left(i\right)}r_{i+1}\right)$, $\alpha_{\left(i\right)}=\sqrt{\alpha_{1(i)}^{2}+1/4}$, $q_{\left(i\right)}=\omega /c_{m(i)}$, $\alpha_{1(i)}=\sqrt{2(c_{22(i)}+c_{23(i)}-c_{12(i)})/c_{11(i)}}$, $c_{m(i)}=\sqrt{c_{11(i)}/\rho_{\left(i\right)}}$, $c_{11(i)}=c_{22(i)}=\frac{E_{\left(i\right)}\left(1-\nu_{\left(i\right)}\right)}{k_{\left(i\right)}}$, $c_{12(i)}=c_{23(i)}=\frac{E_{\left(i\right)}\nu_{\left(i\right)}}{k_{\left(i\right)}}$, $k_{\left(i\right)}=\left(1+\nu_{\left(i\right)}\right)\left(1-2\nu_{\left(i\right)}\right)$.

**Figure 3:**
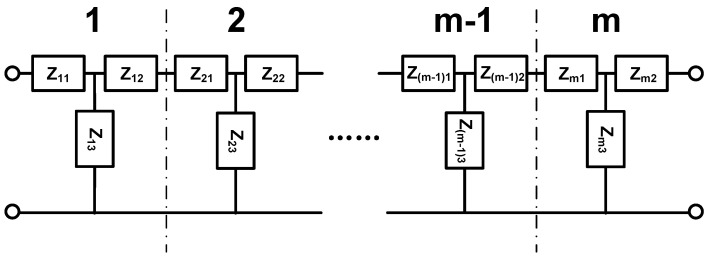
EECM of FGM.

**Figure 4:**
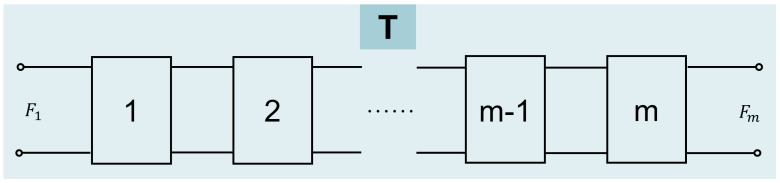
Two-terminal networks of FGM for EECM.

The EECM in Figure 3 can be considered as a cascade of several two-terminal networks, as shown in Figure 4.

According to Figure 4, the relationship between force and vibration velocity can be expressed as

(10) $$\left[\begin{array}{c} F_{i}^{\prime }\\ v_{i}^{\prime } \end{array}\right]=\left[\begin{array}{cc} A_{i} & B_{i}\\ C_{i} & D_{i} \end{array}\right]\left[\begin{array}{c} F_{i+1}^{\prime }\\ -v_{i+1}^{\prime } \end{array}\right],$$

where $A_{i}=\frac{Z_{11i}}{Z_{21i}}$, $B_{i}=\frac{∆Z_{i}}{Z_{21i}}$, $C_{i}=\frac{1}{Z_{21i}}$, $D_{i}=\frac{Z_{22i}}{Z_{21i}}$, $Z_{11i}=Z_{i1}+Z_{i3}$, $Z_{22i}=Z_{i2}+Z_{i3}$, $Z_{12i}=Z_{21i}=Z_{i3}$, $∆Z_{i}=\left|\begin{array}{cc} Z_{11i} & Z_{12i}\\ Z_{21i} & Z_{22i} \end{array}\right|$.

The parameters of the two-terminal network are related as follows,

(11) $$T=\left[\begin{array}{cc} A & B\\ C & D \end{array}\right]=T_{1}T_{2}\ldots T_{m},$$

where $T_{i}=\left[\begin{array}{cc} A_{i} & B_{i}\\ C_{i} & D_{i} \end{array}\right]$.

**Figure 5:**
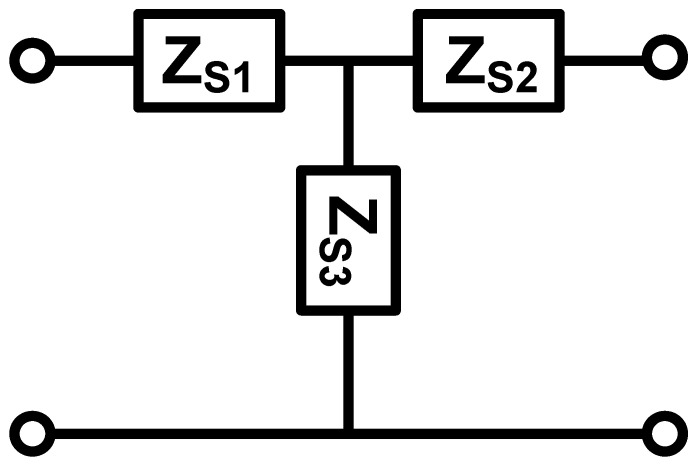
EECM of FGM after simplification.

Therefore, the EECM of FGM can be simplified as shown in Figure 5, where $Z_{S1}=\frac{A}{C}-\frac{∆T}{C}$, $Z_{S2}=\frac{D}{C}-\frac{∆T}{C}$, $Z_{S3}=\frac{∆T}{C}$, $∆T=\left|\begin{array}{cc} A & B\\ C & D \end{array}\right|$.

**Figure 6:**
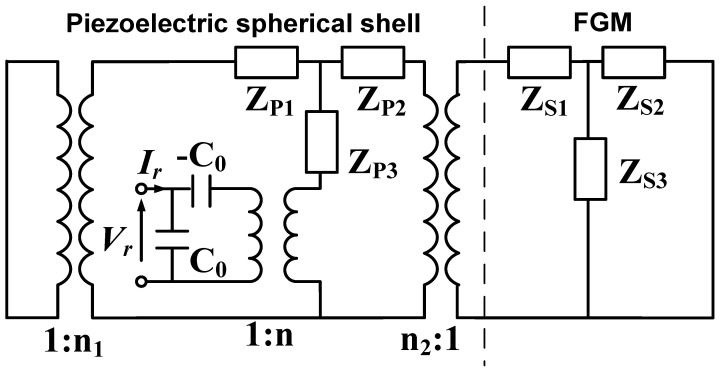
EECM of FGM-cSPT.

Based on the boundary conditions at the interfaces, the electromechanical equivalent circuits of FGM shells and spherical piezoelectric ceramics can be connected in cascaded, parallel series. Similar operations can also be applied when analyzing FGM spherical shells in conjunction with other vibrating systems, which demonstrates the simplicity of using electromechanical equivalent circuit models in processing vibrating systems. According to the continuity of force and vibration velocity at the interface between the FGM spherical shell and the spherical piezoelectric transducer, the EECM of the FGM-cSPT can be obtained, as shown in Figure 6.

Since the derivation process of the EECM of the piezoelectric ceramic spherical shell has been given in detail in reference [5], it will not be repeated in this paper. The impedance expressions of the piezoelectric ceramic spherical shell in the figure are as follows,

(12) $$\begin{array}{c} Z_{p1}=\frac{j4\pi abM_{b}^{2}}{\omega }\left(H_{1}-\frac{1}{2}H_{2}-H_{2}\mu \right)\\ \;+\frac{j{4\pi a^{2}bM_{b}^{2}H}_{2}\lambda }{\omega \Delta_{1}}\left[\begin{array}{c} J_{\mu }\left(\lambda b\right)Y_{\mu -1}\left(\lambda a\right)\\ -Y_{\mu }\left(\lambda b\right)J_{\mu -1}\left(\lambda a\right) \end{array}\right]\\ -\frac{8jH_{2}abM_{a}M_{b}}{\omega \Delta_{1}}, \end{array}$$

(13) $$\begin{array}{c} Z_{p2}=\frac{j4\pi abM_{a}^{2}}{\omega }\left({-H}_{1}+\frac{1}{2}H_{2}+H_{2}\mu \right)\\ +\frac{j{4\pi b^{2}aM_{a}^{2}H}_{2}\lambda }{\omega \Delta_{1}}\left[\begin{array}{c} J_{\mu }\left(\lambda a\right)Y_{\mu -1}\left(\lambda b\right)\\ -Y_{\mu }\left(\lambda a\right)J_{\mu -1}\left(\lambda b\right) \end{array}\right]\\ -\frac{8jH_{2}abM_{a}M_{b}}{\omega \Delta_{1}}, \end{array}$$

(14) $$Z_{p3}=\frac{8jH_{2}abM_{a}M_{b}}{\omega \Delta_{1}},$$

where $M_{a}=\frac{e_{31}\sqrt{\lambda }}{\varepsilon_{33}{∆}_{1}}\left\{\begin{array}{c} -\frac{4}{\pi }s_{-\frac{3}{2},\mu }\left(\lambda b\right)+2a\lambda {∆}_{1}\left(\mu +\frac{5}{2}\right)s_{-\frac{5}{2},\mu +1}\left(\lambda a\right)\\ +2a\lambda \left[\begin{array}{c} J_{\mu +1}\left(\lambda a\right)Y_{\mu }\left(\lambda b\right)\\ -Y_{\mu +1}\left(\lambda a\right)J_{\mu }\left(\lambda b\right) \end{array}\right]s_{-\frac{3}{2},\mu }\left(\lambda a\right) \end{array}\right\}-\frac{e_{33}}{\varepsilon_{33}}a^{-\frac{1}{2}}$, $M_{b}=\frac{e_{31}\sqrt{\lambda }}{\varepsilon_{33}{∆}_{1}}\left\{\begin{array}{c} -\frac{4}{\pi }s_{-\frac{3}{2},\mu }\left(\lambda a\right)+2b\lambda {∆}_{1}\left(\mu +\frac{5}{2}\right)s_{-\frac{5}{2},\mu +1}\left(\lambda b\right)\\ +2b\lambda \left[\begin{array}{c} J_{\mu +1}\left(\lambda b\right)Y_{\mu }\left(\lambda a\right)\\ -Y_{\mu +1}\left(\lambda b\right)J_{\mu }\left(\lambda a\right) \end{array}\right]s_{-\frac{3}{2},\mu }\left(\lambda b\right) \end{array}\right\}-\frac{e_{33}}{\varepsilon_{33}}b^{-\frac{1}{2}}$, $\kappa =\frac{1}{P}$, $P=-\frac{1}{\varepsilon_{33}^{s}}\left(\frac{1}{b}-\frac{1}{a}\right)-\eta \Delta_{4}+\frac{\Delta_{2}\Delta_{5}}{\Delta_{1}}-\frac{\Delta_{3}\Delta_{6}}{\Delta_{1}}$, $\Delta_{4}=2\frac{e_{31}}{\varepsilon_{33}^{s}}\sqrt{\lambda }S+\frac{e_{33}}{\varepsilon_{33}^{s}}\sqrt{\lambda }\left[b^{-\frac{1}{2}}s_{-\frac{3}{2},\mu }\left(\lambda b\right)-a^{-\frac{1}{2}}s_{-\frac{3}{2},\mu }\left(\lambda a\right)\right]$, $\Delta_{5}=2\frac{e_{31}}{\varepsilon_{33}^{s}}\sqrt{\lambda }J+\frac{e_{33}}{\varepsilon_{33}^{s}}\sqrt{\lambda }\left[b^{-\frac{1}{2}}J_{\mu }\left(\lambda b\right)-a^{-\frac{1}{2}}J_{\mu }\left(\lambda a\right)\right]$, $\Delta_{6}=2\frac{e_{31}}{\varepsilon_{33}^{s}}\sqrt{\lambda }Y+\frac{e_{33}}{\varepsilon_{33}^{s}}\sqrt{\lambda }\left[b^{-\frac{1}{2}}Y_{\mu }\left(\lambda b\right)-a^{-\frac{1}{2}}Y_{\mu }\left(\lambda a\right)\right]$, $S=\left.\frac{1}{4\sqrt{\lambda }r\left(\frac{1}{2}\mu +\frac{1}{4}\right)\left(\frac{1}{2}\mu -\frac{1}{4}\right)}{2_{2}F}_{3}\left(\left[-\frac{1}{2},1\right],\left[\frac{1}{2},-\frac{1}{2}\mu +\frac{3}{4},\frac{1}{2}\mu +\frac{3}{4}\right],-\frac{1}{4}r^{2}\lambda^{2}\right)\right|\begin{array}{c} b\\ a \end{array}$, $J=\frac{\sqrt{2}}{4}\sqrt{\lambda }\left.\left\{\frac{\sqrt{2}r^{-\frac{1}{2}}\lambda^{-\frac{1}{2}}\left[4r^{2}\lambda^{2}+\left(2\mu +1\right)\left(3+2\mu \right)\right]J_{\mu }\left(\lambda r\right)}{\left(\frac{1}{2}\mu -\frac{1}{4}\right)\left(2\mu +1\right)\left(3+2\mu \right)}-\frac{2\sqrt{2}r^{\frac{1}{2}}\lambda^{\frac{1}{2}}J_{\mu +1}\left(\lambda r\right)}{\left(\frac{1}{2}\mu -\frac{1}{4}\right)\left(2\mu +1\right)}-\frac{4\sqrt{2}r\lambda J_{\mu }\left(\lambda r\right)s_{\frac{3}{2},\mu }\left(\lambda r\right)}{\left(\frac{1}{2}\mu -\frac{1}{4}\right)\left(2\mu +1\right)\left(3+2\mu \right)}+\frac{2\sqrt{2}r\lambda J_{\mu +1}\left(\lambda r\right)s_{\frac{1}{2},\mu }\left(\lambda r\right)}{\left(\frac{1}{2}\mu -\frac{1}{4}\right)\left(2\mu +1\right)}\right\}\right|\begin{array}{c} b\\ a \end{array}$, $Y=\frac{\sqrt{2}}{4}\sqrt{\lambda }\left.\left\{\frac{\sqrt{2}r^{-\frac{1}{2}}\lambda^{-\frac{1}{2}}\left[4r^{2}\lambda^{2}+\left(2\mu +1\right)\left(3+2\mu \right)\right]Y_{\mu }\left(\lambda r\right)}{\left(\frac{1}{2}\mu -\frac{1}{4}\right)\left(2\mu +1\right)\left(3+2\mu \right)}-\frac{2\sqrt{2}r^{\frac{1}{2}}\lambda^{\frac{1}{2}}Y_{\mu +1}\left(\lambda r\right)}{\left(\frac{1}{2}\mu -\frac{1}{4}\right)\left(2\mu +1\right)}-\frac{4\sqrt{2}r\lambda Y_{\mu }\left(\lambda r\right)s_{\frac{3}{2},\mu }\left(\lambda r\right)}{\left(\frac{1}{2}\mu -\frac{1}{4}\right)\left(2\mu +1\right)\left(3+2\mu \right)}+\frac{2\sqrt{2}r\lambda Y_{\mu +1}\left(\lambda r\right)s_{\frac{1}{2},\mu }\left(\lambda r\right)}{\left(\frac{1}{2}\mu -\frac{1}{4}\right)\left(2\mu +1\right)}\right\}\right|\begin{array}{c} b\\ a \end{array}$, $H_{1}=2c_{13}+2e_{31}e_{33}/\varepsilon_{33}^{s}$, $H_{2}=c_{33}+e_{33}^{2}/\varepsilon_{33}^{s}$, $n_{1}=\sqrt{b}M_{b}$, $n_{2}=\sqrt{a}M_{a}$, $n_{0}=4\pi \sqrt{a}\sqrt{b}{M_{a}M}_{b}\kappa$, $C_{0}=4\pi \kappa$, _m_F_n_ ([a_1_…a_m_], [b_1_…b_m_], x) is the generalized hypergeometric function which is given by hypergeometric series.

According to Figure 6, the input impedance of the transducer is

(15) $$Z_{eq}=\frac{V_{r}}{I_{r}}=\frac{n_{0}^{2}-j\omega C_{0}Z_{m}}{\omega^{2}C_{0}^{2}Z_{m}},$$

where $Z_{m}=Z_{p3}+\frac{Z_{p1}Z_{f}}{Z_{p1}{+Z}_{f}}$ and $Z_{f}=Z_{p2}+n_{2}^{2}(Z_{S1}+\frac{Z_{S2}Z_{S3}}{Z_{S2}{+Z}_{S3}})$.

When the input impedance tends to zero, the resonance frequency equation can be written as

(16) $$n_{0}^{2}-j\omega C_{0}Z_{m}=0.$$

When the input impedance tends to infinity, the antiresonance frequency equation can be obtained as

(17) $$\omega^{2}C_{0}^{2}Z_{m}=0.$$

## 3. Results and Discussion

### 3.1. Vibration Modes and Admittance

**Figure 7:**
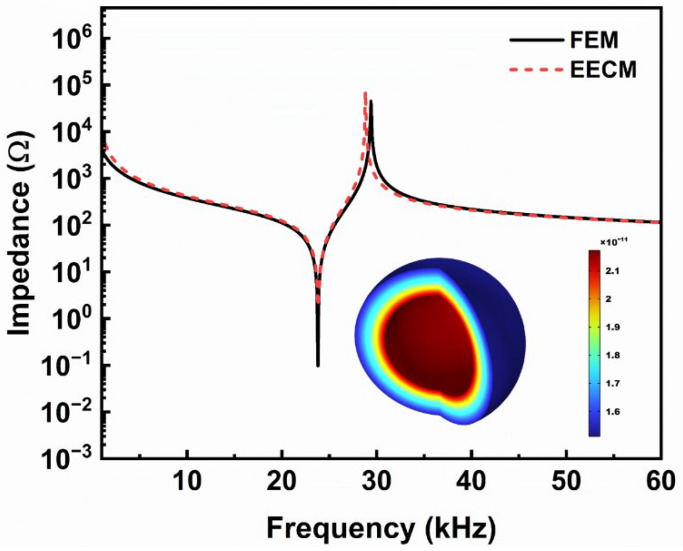
Impedance and first vibration modes of FGM-cSPT.

Since Equations (15) and (16) are transcendental equations, whose analytic solutions cannot be obtained directly, Wolfram Mathematica 11.3 is used to solve the frequency equation in the following analysis. In addition, the numerical simulations were carried out using the FEM based on the solid mechanics and electrostatic modules of the commercial software COMSOL Multiphysics 6.2. Figure 7 shows the impedance and the first radial vibration mode, where the geometric dimensions of the FGM-cSPT are a = 30 mm, b = 35 mm, and c = 36 mm, and the non-uniformity coefficient β = 1. The materials used are shown in Table 1. The materials set up in COMSOL Multiphysics 6.2 are shown in Appendix A. The black line and red dashed line represent the results of the FEM and EECM, respectively, and the admittance obtained using the FEM is in good agreement with the EECM results. The first-mode resonance frequency and anti-resonance frequency of the FGM-cSPT obtained using the EECM are 23,794 Hz and 28,808 Hz, respectively. The first-mode resonance frequency and anti-resonance frequency of the FGM-cSPT obtained using the FEM are 23,798 Hz and 29,376 Hz, respectively. The first radial vibration mode calculated using the FEM is shown in the illustration, which is also known as the breathing vibration mode.

The proposed EECM is effective for analyzing radial vibrations of spherical piezoelectric transducers. However, when more coupled vibrations are involved, more complex models are necessary for accurate analysis.

### 3.2. Effect of FGM on Vibration Characteristics of FGM-cSPT

**Figure 8:**
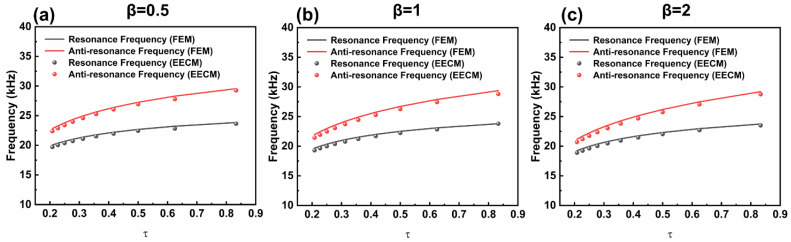
The relationship between the resonance frequency/anti-resonance frequency for the FGM-cSPT and the geometric dimension and non-uniformity coefficient: (**a**) β = 0.5, (**b**) β = 1, and (**c**) β = 2.

As a composite part of a spherical piezoelectric transducer, the FGM layer inevitably affects the overall vibration characteristics of the transducer. As shown in Equations (1)–(3) in Section 2, the non-uniform coefficient β affects the composition of the material. Hence, the effect of the FGM layer regarding thickness and the non-uniformity coefficient (β) on the vibration characteristics of the FGM-cSPT is investigated based on the EECM and FEM, as shown in Figure 8. Here, a dimensionless quantity(τ) is introduced, where *τ*$=\frac{b-a}{c-a}$.

The resonance frequency and anti-resonance frequency obtained using the EECM and FEM are consistent in different geometric dimensions and non-uniform coefficients. As τ increases, the resonance frequency and anti-resonance frequency of the FGM-cSPT increase. In other words, the increase in the wall thickness of the FGM layer leads to a decrease in both the resonance frequency and anti-resonance frequency of the FGM-cSPT, which is consistent with the change in the resonance frequency and anti-resonance frequency of the composite spherical piezoelectric transducer when the outer layer thickness increases. Furthermore, by comparing the resonance frequency and anti-resonance frequency at β = 0.5, 1, and 2, it can be found that as β increases, both the resonance frequency and anti-resonance frequency decrease. Accordingly, the non-uniform coefficient should be considered in engineering design.

**Figure 9:**
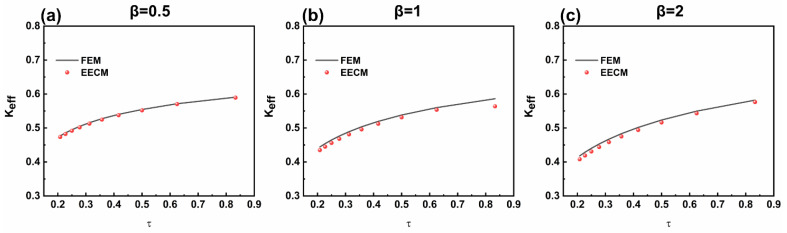
The relationship between the effective electromechanical coupling coefficient of the FGM-cSPT and the geometric dimension and non-uniformity coefficient: (**a**) β = 0.5, (**b**) β = 1, and (**c**) β = 2.

As the value of τ rises, the effective electromechanical coupling coefficient also ascends, as shown in Figure 9.

As the wall thickness of the FGM layer grows, the effective electromechanical coupling coefficient declines. Furthermore, by comparing β = 0.5, 1, and 2, it can be observed that as the β increases, the effective electromechanical coupling coefficient decreases. Therefore, when designing the FGM-cSPT, it is necessary to balance the effective electromechanical coupling coefficient and other performance indicators in the engineering design process. The effective electromechanical coupling coefficient obtained using the EECM and FEM are consistent in different geometric dimensions and non-uniform coefficients.

The FGM-cSPT is anticipated to be utilized in ocean exploration and structural health monitoring due to its omnidirectional radiation and gradient-matching properties. In addition, the great potential of non-uniform composite layers in acoustic impedance matching is also expected to improve the bandwidth of spherical piezoelectric transducers. However, the realization of FGMs that precisely adhere to theoretical specifications presents substantial manufacturing challenges. The theoretical model can be used as a guide for device design, such as the fabrication of heterogeneous models of structural gradient composites rather than materials that strictly conform to the density, Young’s modulus, and Poisson’s ratio functional gradient changes in Equations (1)–(3).

### 3.3. Experiment

Due to the difficulty of manufacturing FGM in practice, the correctness verification of theoretical models for FGM is carried out by degrading them to the theoretical models of isotropic materials to verify their validity [26,27,28]. Therefore, we degenerated the theoretical model for FGM to that for an isotropic material to verify the correctness.

**Figure 10:**
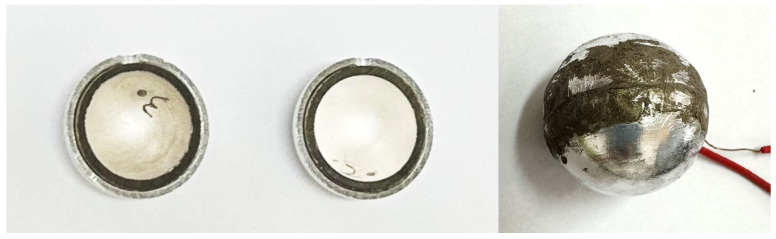
The physical picture of the degraded piezoelectric transducer.

Here, aluminum is selected as the composite layer. Because it is difficult to manufacture the whole spherical shell, the composite spherical piezoelectric transducer comprises two hemispheres. The prototype used in the experiment is shown in Figure 10. An impedance analyzer (BEIJING BAND ERA CO., LTD, PV520A-V, Beijing, China) was used to test the impedance frequency response curve. The results of the theoretical model and FEM were compared with the experimental test results, as shown in Table 2. The piezoelectric ceramic is PZT-4, with an outer diameter of 19.5 mm and an inner diameter of 16.5 mm. The spherical shell of the composite layer is made of aluminum, with an outer diameter of 22.5 mm and an inner diameter of 19.5 mm. The material parameters of PZT-4 and aluminum are shown in Table 3, where $\Delta_{f1}=\left|f_{re}-f_{rf}\right|/f_{re}$, $\Delta_{f2}=\left|f_{ae}-f_{af}\right|/f_{ae}$, $\Delta_{t1}=\left|f_{re}-f_{rt}\right|/f_{re}$, and $\Delta_{t2}=\left|f_{ae}-f_{at}\right|/f_{ae}$.

The resonance and anti-resonance frequencies for the composite spherical piezoelectric transducer are 105,333 Hz and 119,717 Hz based on the experiment, 109,980 Hz and 125,080 Hz based on the FEM, and 109,854 Hz and 125,500 Hz based on the EECM. Comparing the results for the experiment, FEM, and EECM, the results are consistent, which further explains the correctness of the theoretical model. The errors between the FEM and the experimental results are 4.41% for the resonance frequency and 4.48% for the anti-resonance frequency. In comparison, the errors between the EECM and the experimental results are 4.29% for the resonance frequency and 4.83% for the anti-resonance frequency. There are two main reasons for the errors. First, the material parameters used in the theoretical model and FEM model are based on standard values, while the piezoelectric material used in the experiment may have slight variations from these standard values, leading to experimental errors. Second, the theoretical model and FEM model assume a complete spherical shell, whereas the actual model consists of two hemispheres. This difference reduces the overall stiffness and contributes to the experimental error.

## 4. Conclusions

Here, an FGM-cSPT is proposed, and a theoretical model is established. The correctness of the theoretical model is verified using the FEM and by experiment. Based on the theoretical model, the electromechanical vibration characteristics of the FGM-cSPT are analyzed. The results show that the non-uniform coefficient of FGM can realize the regulation of the vibration characteristics of spherical piezoelectric transducers. The increase in the wall thickness of the FGM layer leads to a decrease in both the resonance frequency and anti-resonance frequency of the spherical piezoelectric transducer. When designing an FGM-cSPT, it is necessary to balance the effective electromechanical coupling coefficient and other performance indicators. In addition, the theory is universal and suitable for analyzing typical composite spherical piezoelectric transducers. The theory proposed in this paper can effectively guide the design of an FGM-cSPT and promote the application of the transducer in marine exploitation, structural health detection, and energy collection.

## Figures and Tables

**Table 1:** Material parameters of PZT-5A, ceramic, and hydrogel.

Parameters	Value	
	PZT-5A	
$\rho \;(kg/m^{3})$	7750	
$c_{11}^{E}\;(\times 10^{10}\;N/m^{2})$	12.1	
$c_{12}^{E}\;(\times 10^{10}\;N/m^{2})$	7.54	
$c_{13}^{E}\;(\times 10^{10}\;N/m^{2})$	7.52	
$c_{33}^{E}\;(\times 10^{10}\;N/m^{2})$	11.1	
$e_{31}Nm/V$	−5.4	
$e_{33}Nm/V$	15.8	
$\varepsilon_{33}^{s}\;(\times 10^{-9}\;C/m)$	7.35	
Parameters	Value	
	Ceramic	Hydrogel
$\rho \;(kg/m^{3})$	7700	1070
E (N/m^2^)	53.2 × 10^9^	790
σ	0.26	0.44

**Table 2:** Comparison of experimental, FEM, and theoretical results.

Experiment		FEM				EECM			
*f_re_* (Hz)	*f_ae_* (Hz)	*f_rf_* (Hz)	Δ_f1_%	*f_af_* (Hz)	Δ_f2_%	*f_rt_* (Hz)	Δ_t1_%	*f_at_* (Hz)	Δ_t2_%
105,333	119,717	109,980	4.41	125,080	4.48	109,854	4.29	125,500	4.83

**Table 3:** Material parameters of PZT-4 and aluminum.

Parameters	Value
	PZT-4
$\rho \;(kg/m^{3})$	7500
$c_{11}^{E}\;(\times 10^{10}\;N/m^{2})$	13.9
$c_{12}^{E}\;(\times 10^{10}\;N/m^{2})$	7.78
$c_{13}^{E}\;(\times 10^{10}\;N/m^{2})$	7.43
$c_{33}^{E}\;(\times 10^{10}\;N/m^{2})$	11.5
$e_{31}Nm/V$	−5.2
$e_{33}Nm/V$	15.1
$\varepsilon_{33}^{s}\;(\times 10^{-9}\;C/m)$	5.62
Parameters	Value
	Aluminum
$\rho \;(kg/m^{3})$	2700
E (N/m^2^)	69 × 10^9^
σ	0.33

## Data Availability

The data presented in this study are available on request from the corresponding author due to privacy.

## Abbreviations

The following abbreviations are used in this manuscript:

FEM	Finite element method
EECM	Electromechanical equivalent circuit method
FGM	Functionally graded material
FGM-cSPT	Functionally graded material composite spherical PZT-5A piezoelectric transducer

## Appendix A

In the FEM simulation, the functionally graded piezoelectric material parameters are set as shown below.

**Density:** ρ_c_+(ρ_w_−ρ_c_)×((((R^2+Z^2)^0.5-a)/(b-a))^β)
**Young’s modulus:** E_c_+(E_w_-E_c_)×((((R^2+Z^2)^0.5-a)/(b-a))^β)
**Poisson’s ratio:** v_c_+(v_w_-v_c_)×((((R^2+Z^2)^0.5-a)/(b-a))^β)

where $E_{p}$, $\rho_{p}$, and $\nu_{p}$ are, respectively, Young’s modulus, density, and Poisson’s ratio of ceramics; $E_{W}$, $\rho_{W}$, and $\nu_{W}$ are, respectively, Young’s modulus, density, and Poisson’s ratio of hydrogels.

## References

[1] Zhang Y.J., Wang L.K., Qin L., Zhong C., Hao S.H. (2021). Spherical–Omnidirectional Piezoelectric Composite Transducer for High Frequency Underwater Acoustics. IEEE Trans. Ultrason. Ferroelectr. Freq. Control.

[2] Tang Y.F., Chen C., Wang C.H., Lin S.Y. (2024). A universal analysis method for an omnidirectional broadband spherical transducer based on 1-3-2 piezoelectric composite. Mech. Syst. Signal. Prac..

[3] Ma K.J., Chen H.Y., Wu Z.Y., Hao X.L., Ya G., Li W.B., Shao L., Meng G., Zhang W.M. (2022). A wave-confining metasphere beamforming acoustic sensor for superior human-machine voice interaction. Sci. Adv..

[4] Wang S., Lin S.Y. (2021). A novelly universal theory: Toward accurately evaluating radial vibration characteristics for radially sandwiched spherical piezoelectric transducer. Ultrasonics.

[5] Wang S., Lin S.Y. (2021). An Exact and Practical Analyzing Model for Radial Vibration of Piezoelectric Spherical Transducers With Arbitrary Wall Thickness. IEEE Trans. Ultrason. Ferroelectr. Freq. Control.

[6] Tang Y.F., Chen C., Tian H., Lin S.Y. (2024). Radial vibration analysis for piezoceramic shell-stacked spherical transducer with thick walls. Smart Mater. Struct..

[7] Tang Y.F., Lin S.Y. (2023). Systematic design and experimental realization of a radially cascaded spherical piezoelectric transducer. J. Acoust. Soc. Am..

[8] Kong Q.Z., Fan S.L., Bai X.L., Mo Y.L., Song G.B. (2017). A novel embeddable spherical smart aggregate for structural health monitoring: Part I. Fabrication and electrical characterization. Smart Mater. Struct..

[9] Kong Q.Z., Fan S.L., Mo Y.L., Song G.B. (2017). A novel embeddable spherical smart aggregate for structural health monitoring: Part II. Numerical and experimental verifications Smart Mater. Struct..

[10] Dong E.Q., Song Z.C., Zhang Y., Mosanenzadeh S.G., He Q., Zhao X.H., Fang N.X. (2020). Bioinspired metagel with broadband tunable impedance matching. Sci. Adv..

[11] Bian J.C., Wang Y., Liu Z.J., Shen M.J., Zhao H., Sun Y.L., Zhu J.J. (2021). Ultra-wideband underwater acoustic transducer with a gradient impedance matching layer. Appl. Acoust..

[12] Ji H.W., Qi A.Q., Yang F., Wu X., Lv B., Ni J. (2023). Design of acoustic impedance gradient matching layers. Appl. Acoust..

[13] Zhang Y.W., Chen W.J., Ni Z.Y., Zang J., Hou S. (2020). Supersonic aerodynamic piezoelectric energy harvesting performance of functionally graded beams. Compos. Struct..

[14] Larkin K., Abdelkefi A. (2019). Neutral axis modeling and effectiveness of functionally graded piezoelectric energy harvesters. Compos. Struct..

[15] Foroutan M., Mohammadi F., Alihemati J., Soltanimaleki A. (2017). Dynamic analysis of functionally graded piezoelectric cylindrical panels by a three-dimensional mesh-free model. J. Intel. Mat. Syst. Str..

[16] Zhang X., Li H.G., Xiong H.L., Sun S.T., Ling Z.C., Shen G.D., Zhao X., Chen B.J., Meng G. (2024). Design and experimental validation of a broadband multi-modal coupled capsule-shaped transducer for underwater acoustics. Sens. Actuators A Phys..

[17] Naghdi M., Zhang H.F., Sreedharan S.V., Ju S., Desai M.H. (2024). A new FEM-based approach on the modeling of stress-induced velocity shift of piezoelectric surface acoustic wave resonators. Ultrasonics.

[18] Yamada K., Ieiri S., Itoh S., Kasashima T., Morita T. (2024). Rayleigh wave excitation with an elliptical reflector for high-power ultrasound. Sens. Actuators A Phys..

[19] Yang T.Y., Zhu Y.F., Li S.Y., An D.W., Yao M., Cao W.W. (2020). Dielectric loss and thermal effect in high power piezoelectric systems. Sens. Actuators A Phys..

[20] Maadi M., Zemp R.A. (2019). Nonlinear Lumped Equivalent Circuit Model for a Single Uncollapsed Square CMUT Cell. J. IEEE Trans. Ultrason. Ferroelectr. Freq. Control.

[21] Shim H., Roh Y. (2021). Development of an equivalent circuit of a cymbal transducer. IEEE Sens. J..

[22] Chen D.D., Zhao J.X., Fei C.L., Li D., Zhu Y.B., Li Z.X., Guo R., Lou L.F., Feng W., Yang Y.T. (2020). Particle Swarm Optimization Algorithm-Based Design Method for Ultrasonic Transducers. Micromachines.

[23] Pyo S., Lim Y., Roh Y. (2021). Analysis of the transmitting characteristics of an acoustic conformal array of multimode tonpilz transducers by the equivalent circuit method. Sens. Actuators A Phys..

[24] Hasheminejad S.M., Malakooti S., Akbarzadeh H.M. (2011). Acoustic radiation from a submerged hollow FGM sphere. Arch. Appl. Mech..

[25] Hasheminejad S.M., Gudarzi M. (2015). Active sound radiation control of a submerged piezocomposite hollow sphere. J. Intell. Mater. Syst. Struct..

[26] Wang S., Chen C., Hu L.Q., Lin S.Y. (2022). Spherical piezoelectric transducers of functionally graded materials. J Acoust. Soc. Am..

[27] Wang S., Shan J.J., Lin S.Y. (2022). Radial vibration analysis for functionally graded ring piezoelectric transducers based on electromechanical equivalent circuit method. Ultrasonics.

[28] Wang H.M., Luo D.S. (2015). Exact analysis of radial vibration of functionally graded piezoelectric ring transducers resting on elastic foundation. Appl. Math. Model.

